# Cost–effectiveness of results-based financing, Zambia: a cluster randomized trial

**DOI:** 10.2471/BLT.17.207100

**Published:** 2018-08-29

**Authors:** Wu Zeng, Donald S Shepard, Ha Nguyen, Collins Chansa, Ashis Kumar Das, Jumana Qamruddin, Jed Friedman

**Affiliations:** a Schneider Institutes for Health Policy, The Heller School, MS 035, Brandeis University, Waltham, Massachusetts 02454-9110, United States of America (USA).; b Health, Nutrition and Population Global Practice, The World Bank Group, Washington DC, USA.; c Institute of Global Health, Heidelberg University, Heidelberg, Germany.; d Development Research Group, The World Bank Group, Washington DC, USA.

## Abstract

**Objective:**

To evaluate the cost–effectiveness of results-based financing and input-based financing to increase use and quality of maternal and child health services in rural areas of Zambia.

**Methods:**

In a cluster-randomized trial from April 2012 to June 2014, 30 districts were allocated to three groups: results-based financing (increased funding tied to performance on pre-agreed indicators), input-based financing (increased funding not tied to performance) or control (no additional funding), serving populations of 1.33, 1.26 and 1.40 million people, respectively. We assessed incremental financial costs for programme implementation and verification, consumables and supervision. We evaluated coverage and quality effectiveness of maternal and child health services before and after the trial, using data from household and facility surveys, and converted these to quality-adjusted life years (QALYs) gained.

**Findings:**

Coverage and quality of care increased significantly more in results-based financing than control districts: difference in differences for coverage were 12.8% for institutional deliveries, 8.2% postnatal care, 19.5% injectable contraceptives, 3.0% intermittent preventive treatment in pregnancy and 6.1% to 29.4% vaccinations. In input-based financing districts, coverage increased significantly more versus the control for institutional deliveries (17.5%) and postnatal care (13.2%). Compared with control districts, 641 more lives were saved (lower–upper bounds: 580–700) in results-based financing districts and 362 lives (lower–upper bounds: 293–430) in input-based financing districts. The corresponding incremental cost–effectiveness ratios were 809 United States dollars (US$) and US$ 413 per QALY gained, respectively.

**Conclusion:**

Compared with the control, both results-based financing and input-based financing were cost–effective in Zambia.

## Résumé

**Objectif:**

Évaluer le rapport coût-efficacité du financement axé sur les résultats et du financement axé sur les apports pour accroître l'utilisation et la qualité des services de santé maternelle et infantile dans les zones rurales de Zambie.

**Méthodes:**

Dans un essai randomisé par grappes mené d'avril 2012 à juin 2014, 30 districts ont été répartis en trois groupes: financement axé sur les résultats (hausse du financement liée aux performances vis-à-vis d'indicateurs préalablement définis), financement axé sur les apports (hausse du financement non liée aux performances) et groupe témoin (pas de financement supplémentaire), qui représentaient respectivement une population de 1,33, 1,26 et 1,40 millions de personnes. Nous avons estimé les coûts financiers supplémentaires de la mise en œuvre et de la vérification des programmes, des consommables et de la supervision. Nous avons évalué la couverture et la qualité des services de santé maternelle et infantile avant et après l'essai, à l'aide de données provenant d'enquêtes auprès des ménages et des établissements, et les avons converties en années de vie ajustées par la qualité (AVAQ) gagnées.

**Résultats:**

La couverture et la qualité des soins ont nettement plus augmenté dans les districts recevant un financement axé sur les résultats que dans les districts témoins: la différence des différences en matière de couverture était de 12,8% pour les accouchements dans des structures médicales, de 8,2% pour les soins postnataux, de 19,5% pour les contraceptifs injectables, de 3,0% pour les traitements préventifs intermittents pendant la grossesse et de 6,1% à 29,4% pour les vaccinations. Dans les districts recevant un financement axé sur les apports, la couverture a nettement plus augmenté que dans les districts témoins pour ce qui est des accouchements dans des structures médicales (17,5%) et des soins postnataux (13,2%). Par rapport aux districts témoins, 641 vies supplémentaires ont été sauvées (bornes inférieure-supérieure: 580–700) dans les districts recevant un financement axé sur les résultats et 362 vies (bornes inférieure-supérieure: 293–430) dans les districts recevant un financement axé sur les apports. Les ratios coût-efficacité correspondants étaient respectivement de 809 dollars des États-Unis ($ US) et 413 $ US en plus par AVAQ gagnée.

**Conclusion:**

Comparés au groupe témoin, le financement axé sur les résultats et le financement axé sur les apports présentaient tous deux un rapport coût-efficacité positif en Zambie.

## Resumen

**Objetivo:**

Evaluar la relación entre el gasto y la efectividad de la financiación basada en los resultados y la financiación basada en insumos para aumentar el uso y la calidad de los servicios de salud materno-infantil en las zonas rurales de Zambia.

**Métodos:**

En un ensayo aleatorio por conglomerados de abril de 2012 a junio de 2014, se asignaron 30 distritos a tres grupos: financiamiento basado en resultados (aumento de fondos ligados al desempeño en indicadores previamente acordados), financiamiento basado en insumos (aumento de fondos no vinculados a rendimiento) o control (sin financiación adicional), atendiendo poblaciones de 1,33, 1,26 y 1,40 millones de personas, respectivamente. Evaluamos los gastos financieros incrementales para la implementación y verificación del programa, los consumibles y la supervisión. Evaluamos la cobertura y la efectividad de la calidad de los servicios de salud materno-infantil antes y después del ensayo, utilizando datos de encuestas domiciliarias y de establecimientos, y los convertimos en años de vida ajustados por calidad (QALY) obtenidos.

**Resultados:**

La cobertura y la calidad de la atención aumentaron más significativamente en el financiamiento basado en resultados que en los distritos de control: la diferencia de diferencias para la cobertura fue 12,8% para partos institucionales, 8,2% para el cuidado postnatal, 19,5% para anticonceptivos inyectables, 3,0% para el tratamiento preventivo intermitente en embarazo y de 6,1% a 29,4% para vacunas. En los distritos de financiación basados ​​en insumos, la cobertura aumentó más significativamente en comparación con el control para los partos institucionales (17,5%) y la atención posnatal (13,2%). En comparación con los distritos de control, se salvaron 641 vidas más (límites inferiores-superiores: 580-700) en distritos de financiamiento basado en resultados y 362 vidas (límites inferiores-superiores: 293-430) en distritos de financiamiento basados ​​en insumos. Las correspondientes ratios incrementales entre gasto y efectividad fueron de 809 dólares estadounidenses (USD) y de 413 USD por AVA, respectivamente.

**Conclusión:**

En comparación con el control, tanto el financiamiento basado en resultados como el basado en insumos fueron rentables en Zambia.

## ملخص

**الغرض:**

تقييم فعالية تكلفة التمويل القائم على النتائج والتمويل القائم على المدخلات لزيادة استخدام خدمات الأمومة والطفولة الصحية وجودتها في المناطق القروية بزامبيا

**الطريقة:**

في تجربة عنقودية معشاة استمرت من أبريل/نيسان 2012 إلى يونيو/حزيران 2014، قسم البنك الدولي 30 مقاطعة إلى ثلاث مجموعات: تمويل قائم على النتائج (حيث ترتبط زيادة التمويل بالأداء القائم على مؤشرات مُتفق عليها مُسبقًا)، أو تمويل قائم على المدخلات (حيث لا يوجد ارتباط بين زيادة التمويل والأداء) أو التحكم (لا يوجد تمويل إضافي)، وذلك لخدمة مجتمعات سكانية يبلغ تعدادها 1.33، و1.26 و1.40 مليون نسمة، على الترتيب. حيث قمنا بتقدير التكاليف المالية الإضافية اللازمة لتطبيق البرنامج والتحقق من المواد الاستهلاكية والإشراف. وقمنا بتقييم تغطية خدمات الأمومة والطفولة الصحية وفعالية جودتها قبل التجربة وبعدها، باستخدام البيانات المستخرجة من الاستقصاءات المنزلية والخدمية، وتحويل هذه البيانات إلى عدد سنوات عمر معدلة بحسب جودة الحياة (QALYs) المكتسبة.

**النتائج:**

تصاعدت التغطية والجودة بشكل مميز أكثر في التمويل القائم على النتائج عن مقاطعات التحكم: فرق الاختلافات للتغطية كان 12.8% للولادة بالمؤسسات، 8.2% للرعاية بعد الولادة، و19.5% لوسائل منع الحمل التي تؤخذ بالحقن، و3.0% العلاج الوقائي الدوري أثناء الحمل و6.1% إلى 29.4% للتحصينات. في مقاطعات التمويل القائم على المدخلات، تصاعدت التغطية بشكل مميز أكثر مقابل التحكم بالنسبة للولادة بالمؤسسات (17.5%) والرعاية بعد الولادة (13.2%). مقارنةً بمقاطعات التحكم، حيث تم إنقاذ 641 شخصاً إضافياً (الحدود الدنيا-العليا: 580-700) في مقاطعات التمويل القائم على النتائج و362 شخصاً (الحدود الدنيا-العليا: 293-430) في مقاطعات التمويل القائم على المدخلات. وكانت معدلات فعالية التكلفة الإضافية 809 دولار أمريكي (US$) و413 دولار أمريكي لكل عدد سنوات عمر معدلة بحسب جودة الحياة المكتسبة على الترتيب.

**الاستنتاج:**

مقارنةً بالتحكم، كانت نتائج كلاً من التمويل القائم على النتائج والتمويل القائم على المدخلات أكثر فعالية من حيث التكلفة في زامبيا.

## 摘要

**目标:**

旨在评估基于结果融资和基于投入融资的成本效益，以提高赞比亚农村地区妇幼保健服务的使用率和质量。

**方法:**

在 2012 年 4 月至 2014 年 6 月的一项集群随机化试验中，30 个地区被划分为三组：基于结果融资（增加资金与拟议指标的绩效挂钩）、基于投入融资（增加资金未与绩效捆绑）或对照组（无额外资金），服务人群分别为 133 万、126 万和 140 万。我们评估了项目实施和验证、消耗品和监督的增量财务成本。我们使用来自家庭和机构的调查数据，评估了试验前后的母婴健康服务的覆盖率和质量有效性，并将这些数据转换为获得的质量调整寿命年 (QALYs)。

**结果:**

基于结果融资的覆盖率和护理质量明显高于对照组：住院分娩覆盖率的双重差分分析为 12.8％，产后护理为 8.2％，注射避孕药为 19.5％，妊娠间歇性预防性治疗为 3.0％，接种疫苗为 6.1％ 至 29.4％。与对照组相比，在基于投入融资的地区，住院分娩 (17.5％) 和产后护理 (13.2％) 的覆盖率显著增加。与对照组相比，基于结果融资的地区挽救了 641 条生命（下限-上限：580-700），基于投入融资的地区挽救了 362 条生命（下限-上限：293-430）。相应的增量成本效益比率分别为每质量调整寿命年增加 809 美元 (US$) 和 413 美元 (US$)。

**结论:**

与对照组相比，基于结果融资和基于投入融资在赞比亚均具有成本效益。

## Резюме

**Цель:**

Оценка рентабельности финансирования на основе результатов и затратного финансирования для улучшения использования и повышения качества медицинского обслуживания матерей и детей в сельской местности Замбии.

**Методы:**

В кластерном рандомизированном исследовании, проводившемся с апреля 2012 г. по июнь 2014 г., 30 районов были поделены на три группы: финансирование по результатам (увеличение финансирования по результатам деятельности согласно предварительно согласованным показателям), затратное финансирование (увеличение финансирования, не зависящее от результатов деятельности) и контрольная группа (отсутствие дополнительного финансирования). Эти группы подразумевали обслуживание 1,33; 1,26 и 1,40 млн человек соответственно. Была проведена оценка дополнительных финансовых расходов, связанных с реализацией и контролем выполнения программы, расходными материалами и надзором. Авторы проанализировали охват населения, а также качество и эффективность медицинского обслуживания матерей и детей до и после проведения исследования, используя данные, полученные в ходе опросов семейств и медицинских учреждений, и получили показатель продолжительности жизни, скорректированный по качеству (QALY).

**Результаты:**

Охват населения и качество медицинского обслуживания значительно возросли в группе финансирования по результатам в сравнении с районами контрольной группы: разница различий в охвате населения составила 12,8% для родов в медицинских учреждениях, 8,2% для послеродового ухода, 19,5% для инъекционных контрацептивов, 3,0% для периодического профилактического лечения во время беременности и от 6,1 до 29,4% для вакцинаций. В районах группы затратного финансирования охват населения медицинским обслуживанием значительно увеличился в сравнении с контрольной группой для родов в медицинских учреждениях (17,5%) и послеродового ухода (13,2%). В сравнении с районами контрольной группы в районах группы финансирования по результатам было спасено на 641 жизнь больше (нижняя-верхняя границы: 580–700), а в районах группы затратного финансирования — на 362 жизни больше (нижняя-верхняя границы: 293–430). Соответствующие коэффициенты прироста рентабельности составили соответственно 809 и 413 долларов США для полученных показателей продолжительности жизни, скорректированных по качеству (QALY).

**Вывод:**

Группы финансирования по результатам и группы затратного финансирования оказались более рентабельными в сравнении с контрольной группой в Замбии.

## Introduction

Over the last two decades, Zambia’s health indicators have improved substantially. Between 1990 and 2013, the under-five mortality rate dropped from 193 to 87 deaths per 1000 live births, and the maternal mortality rate fell from 580 to 280 deaths per 100 000 births.^1^ There has been a substantial reduction in mortality from human immunodeficiency virus and acquired immune deficiency syndrome (HIV/AIDS) and malaria.^2^

Despite these improvements, the under-five mortality rate and maternal mortality rate in Zambia (population 15.15 million in 2013) remain high by regional and international standards. Furthermore, the use of key maternal and child health services remains low and inequitable.^2^ The country faces great challenges in financing the health system, and delivering quality and equitable health services.^3^^,^^4^

Results-based financing, an approach to incentivize providers to improve the quality and quantity of health services, has been implemented in many low- and middle-income countries.^5^ Despite mixed results,^6^^,^^7^ results-based financing has generally shown promise as a way to address maternal and child health concerns and catalyse health-care reforms.^8^^–^^13^ These findings prompted Zambia to initiate a results-based financing programme. With partial funding from the World Bank, Zambia implemented a project in 2008 in Katete district as a pre-pilot site, trying to realign payment to outputs rather than inputs.

Given the promising results from the pre-pilot project, results-based financing in Zambia was expanded to 10 additional districts under the pilot phase in April 2012, covering a population of 1.33 million. The programme design was a contract-in model, whereby health centres were contracted to deliver a specified package of essential maternal and child health services. Once they fulfilled their obligation, payments were made using predefined maternal and child health and quality indicators.^14^ The pilot programme ended in October 2014.

Although many results-based financing programmes have incorporated impact evaluations, the majority of these have focused on use of services. To our knowledge, few publications have systematically combined the cost and health impact to estimate the value for money of results-based financing.^6^^,^^15^ Given increasing demand for health services, governments face financial constraints in funding programmes. This study aimed to evaluate both the cost and health outcomes of Zambia’s results-based financing programme against two counterfactual policies: enhanced financing not explicitly tied to performance (input-based financing) and the existing system of funding without additional financing (control).

## Methods

### Research design

The study used a triplet-matched cluster randomized design to evaluate the impact on health service delivery in a total of 30 districts from eight provinces. District-level data were collected and scores were assigned to districts for population health, socioeconomic conditions and health governance capacity. In most provinces, three districts at or near the provincial median index score in that province were selected and then randomly allocated among the three different groups: results-based financing, input-based financing or control. Health facilities under results-based financing received incentives tied to performance on pre-agreed indicators and were required to use at least 40% of the incentive payment for operational activities. A maximum of 60% of the incentive payment could be used for staff bonuses. Facilities under input-based financing were intended to receive approximately the same amount of funding as those in results-based financing districts, but their funding was not tied to performance. The payment received was used only for operational activities. Given this additional funding and potential expectation of joining the results-based financing programme in the future, health facilities in the input-based financing group could organize themselves for better service provision. Control facilities represented the existing financing method in Zambia, receiving no additional funds. There were 175, 173 and 175 primary health-care facilities in the results-based financing, input-based financing and control groups, serving populations of 1.33, 1.26 and 1.40 million people, respectively. 

**Fig. 1:**
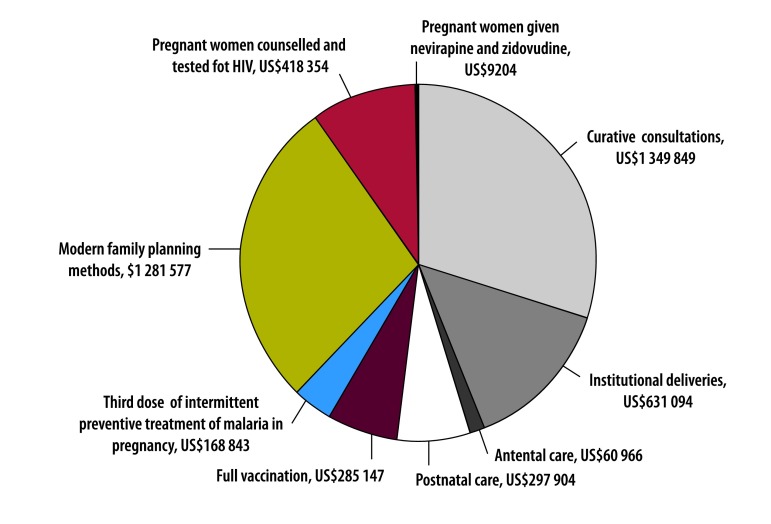
Distribution of payments for nine incentivized services during the implementation period of the results-based financing programme in Zambia, April 2012 to June 2014

HIV: human immunodeficiency virus; US$: United States dollars.

Note: The denominator is US$ 4.50 million of incentive payments to the results-based financing facilities.

Health facilities under results-based financing received incentive payments based on the quantity of nine health services (Box 1) and the quality of 10 aspects of care. Fig. 1 shows the distribution of the incentive payments by service.

Box 1 **Incentivized services (indicators) and incentive payments per service in the results-based financing programme in Zambia**

• Curative consultation: US$ 0.20

• Institutional delivery by skilled birth attendant: US$ 6.40

• Antenatal care, prenatal and follow-up visit: US$ 1.60

• Postnatal care visit: US$ 3.30

• Full immunization of child younger than 1 year: US$ 2.30

• Pregnant woman receiving three doses of malaria intermittent preventive treatment: US$ 1.60

• Family planning user of modern contraceptive method: US$ 0.60

• Pregnant woman counselled and tested for HIV: US$ 1.80

• HIV-positive pregnant woman given nevirapine and zidovudine: US$ 2.00

HIV: human immunodeficiency virus; US$: United States dollars.Source: Government of Zambia, 2011.^14^

### Incremental cost assessment

To provide practical recommendations for decision-making, we conducted the cost–effectiveness analysis from a health-system perspective and examined financial costs rather than economic costs that capture additional opportunity costs. In this analysis, we focused on the incremental cost (additional cost) incurred in the results-based financing and input-based financing groups, compared with the control. Thus, for the cost analysis, the additional costs that we included in the analysis were: the costs of consumables (e.g. drugs and supplies) due to increased services; the costs incurred at World Bank headquarters for designing, implementing and monitoring the programme; and the field costs of implementing the programme in Zambia. All costs were measured in United States dollars (US$) over the period of the programme’s implementation from April 2012 through June 2014. 

We obtained the costs of consumables from a data set compiled by Medical Stores Limited, the national pharmaceutical distribution hub, from January 2011 through June 2014. The cost of consumables for each health facility was calculated as the cost per capita per quarter and then a difference-in-differences approach, which adjusted the baseline differences, was used to determine the incremental cost of consumables. We allocated the World Bank headquarters’ costs to the results-based financing and input-based financing groups in proportion to the implementation costs of each group. We obtained the programme costs from the World Bank Zambia office. Local costs were primarily for administration of the programme (e.g. costs of operations, capacity-building, verification, and monitoring and evaluation) and incentive or facility payments for both results-based financing and input-based financing groups. We converted capital costs (e.g. vehicles) to annual rates, assuming a useful life of 5 years. Given the difference in population size covered by health facilities among the three groups, the programme costs and World Bank headquarters’ costs were calculated as costs per capita.

### Incremental effectiveness assessment

#### Use of health services

To assess the impact of results-based financing on service delivery, the World Bank conducted household surveys before and after the implementation of results-based financing in November to December 2011 (baseline) and November 2014 to January 2015 (endline), respectively. These surveys included 3064 and 3500 households, respectively, with women who had had at least one birth within 2 years before the survey. The household survey results provided estimates of coverage of services for antenatal care, postnatal care, institutional delivery, immunization and use of intermittent preventive treatment for pregnant women.^16^

An impact evaluation was conducted by the World Bank, using the household surveys and a difference-in-differences approach, to examine the effect of results-based financing on key maternal and child health services.^16^ Key advantages of using difference-in-differences analysis are its adjustment for the baseline differences among the three groups, and robustness in estimating the impact.^17^ From the household survey, the following services were included: antenatal care, postnatal care, institutional delivery, intermittent preventive treatment for pregnant women and immunizations. 

At the same time as the household surveys, the World Bank also made health-facility surveys, each of which covered 176 and 210 representative health facilities at the baseline and endline and were verified by field supervisors. For this study, the health-facility survey provided information on use of injectable contraceptives, the general quality of care and service-specific quality measures for constructing quality indexes. We used the use of injectable contraceptives for impact evaluation, after converting the measure to population coverage, as this was the only family planning service showing any statistically significant differences. The calculated national coverage of injectable contraceptives was 21.9% (62 439/285 113) in 2014, similar to the national estimate of 19.3% (1903/9859) in 2013.^2^ The quality measures used by the programme for incentive payments were not used for impact analysis, as they were assessed only for facilities receiving results-based financing. 

Curative services and HIV/AIDS services, not available in the household survey, were not statistically significant based on the data from the health-facility survey, and the Lives Saved Tool could not handle the analysis of the curative care. These two services were therefore excluded from the analysis, but all other incentivized services were included.

#### Quality of health services

**Fig. 2:**
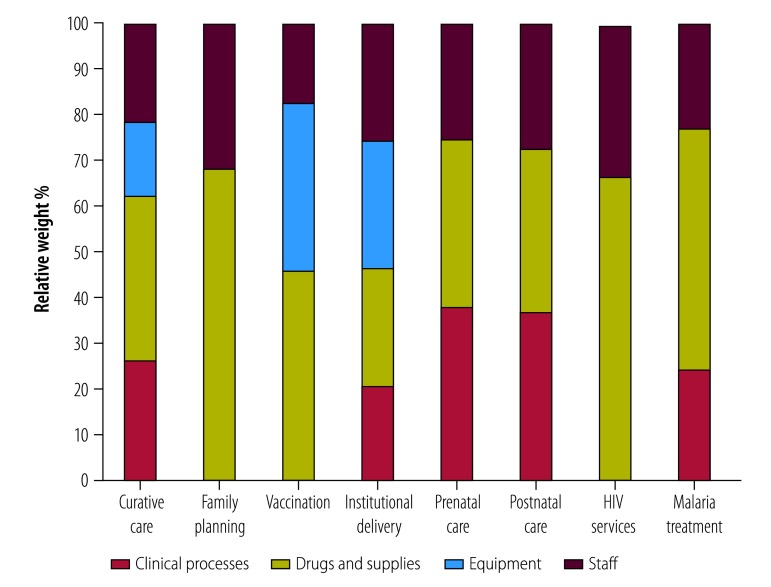
Relative importance of quality components for generating the quality index for each service in the results-based financing programme in Zambia, April 2012 to June 2014

The health-facility survey measured (i) general quality, (ii) clinical process, (iii) availability of drugs and supplies, (iv) equipment and (v) qualified human resources. We selected questions on these five categories from the health-facility survey. We convened a Delphi panel with expertise in epidemiology and clinical medicine in Zambia in November 2014 to determine the relative importance of each quality component, and generated a quality index (ranging from 0 to 1) for each service. Fig. 2 shows the relative importance of each quality component by service. The score of the constructed quality index was similar to that developed from items measuring process quality for the incentive payments in results-based financing facilities.

HIV: human immunodeficiency virus.

Note: Data for some quality components were not available for some services.

Quality of care may not have a linear relationship with health benefit gained from the care (e.g. 80% quality of care does not necessarily mean the care will gain 80% of its full potential of health benefits). To ascertain the impact of quality of care on potential health benefits from the care, we used the same expert panel to generate a health-effect index of quality of care using a quadratic function. The health-effect index estimated how much compromised quality affected the health benefits of a service, expressed as a percentage of the full potential of health benefits when the service was provided optimally. The quadratic function was used, because of its flexibility to accommodate different relationships between variables.

#### Modelling outcomes

We generated a measure of effective coverage by multiplying the health-effect index by the coverage of corresponding services. The result was treated as quality-adjusted coverage to feed into the Lives Saved Tool.^18^^–^^21^ The tool is widely used to estimate maternal and child health outcomes (mortality), with good validity.^22^ However, it only handles a set number of interventions. The tool cannot deal with morbidity, nor can it implement probabilistic sensitivity analyses. We used key parameters from the Zambia data preloaded in the tool and adjusted the population size to that covered by results-based financing. The tool converts the coverage of health services to the number of lives saved from improved services. We converted lives saved into quality-adjusted life years (QALYs), applying the formula for fatal cases^23^ in Equation 1:
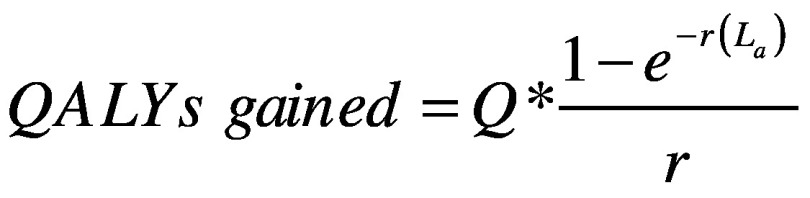
(1)Where *Q* is average quality of life if one survives, *r* is the annual discount rate (we applied 3% in the analysis) and *L_a_* is life expectancy at the age of death of *a*, estimated from Zambia’s life table.^24^

*Q* was estimated from the disease burden in Zambia^25^ by Equation 2 as:
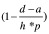
(2)Where *d* is disability, *a* is adjusted life years due to morbidity, *h* is health life expectancy and *p* is population size. 

Years gained in early life play a more important role in determining QALYs. We estimated total QALYs gained by multiplying the number of cases saved by QALYs gained per case. The QALYs gained were then rescaled by the population covered by health facilities to estimate QALYs gained per capita.

### Cost–effectiveness analysis

Based on incremental costs and effectiveness per capita, we estimated the incremental cost–effectiveness ratio under two scenarios: one without quality improvement and the other with it.

To examine the uncertainty of incremental cost–effectiveness ratios, we focused on the uncertainty of the impact of results-based financing on use of health services. To be conservative (i.e. having wider uncertainty intervals) and for the ease of conducting sensitivity analyses, we assumed the independence of the eight indicators included in the analysis. Thus, we used 31.2% confidence intervals (CI) of each indicator to estimate the 95% CI of incremental cost–effectiveness ratios.

While thresholds of cost–effectiveness analysis have been debated,^26^^–^^28^ a study evaluating returns on investment specific to maternal and child health valued a healthy life year as 1.5 times gross domestic product (GDP) per capita,^19^ which we used as the threshold to interpret the results. In 2013, GDP per capita was US$ 1759 in Zambia,^29^ and thus the threshold was estimated at US$ 2639. Interventions with incremental cost–effectiveness ratios smaller than the threshold were regarded as cost–effective.

## Results

**Fig. 3:**
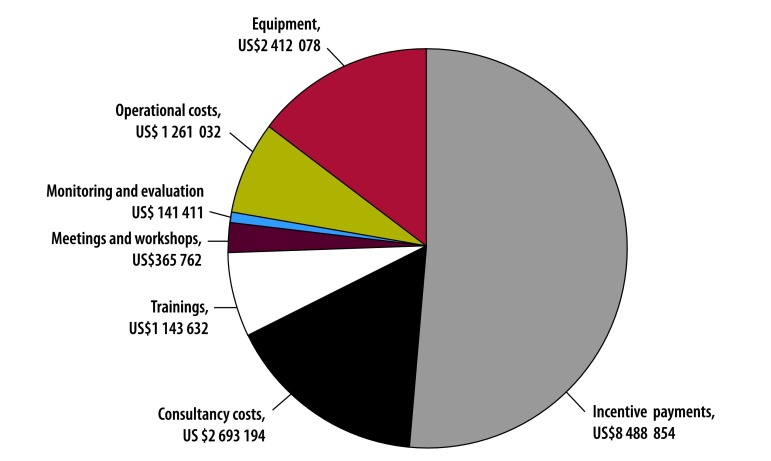
Distribution of costs in the results-based financing programme in Zambia, April 2012 to June 2014

Over the 2.25 years of the programme, costs after annualization were US$ 13.26 million in total, of which US$ 10.54 million (US$ 7.91 per capita) was used in the results-based financing districts, while US$ 2.72 million (US$ 2.16 per capita) was used in the input-based financing districts. Fig. 3 presents the distribution of non-annualized results-based financing and input-based financing programme costs by function. About half of the cost (US$ 8.49 million of the US$16 505 963) was used to pay incentives for results-based financing facilities and to provide comparable funding to input-based financing facilities. The World Bank headquarters’ costs were US$ 566 711, with US$ 450 427 (i.e. US$ 0.34 per capita) and US$ 116 284 (i.e. US$ 0.09 per capita) for the results-based financing and input-based financing groups, respectively.

US$: United States dollars.

Notes: Incentive payments include payments for incentivized services in the results-based financing group and payments to health facilities in the input-based financing group. The denominator is US$ 16.50 million of the overall implementation costs of results-based financing and input-based financing. This number is different from the US$ 13.26 million reported in the main text, because the latter number considers the annualization of capital costs and removes the cost for the pre-pilot project.

The drug and supply costs per capita per quarter in the period before implementation of the programme were estimated at US$ 0.26 for the results-based financing group, US$ 0.50 for the input-based financing and US$ 0.42 for the control (Table 1). These numbers increased to US$ 0.59, US$ 0.77 and US$ 0.64 in the period after programme implementation. Difference in differences estimated that costs of the results-based financing group were US$ 0.57 and US$ 0.97 more than the input-based financing and control groups, respectively, over the implementation period of the programme (9 quarters; Table 2).

**Table 1:** Expenditure on consumables under three methods of health-care financing before and after implementation of the results-based financing programme in Zambia, April 2012 to June 2014

Group	Population in millions	Expenditure, US$			Expenditure per quarter per capita, US$		
		Before programme (5 quarters)	After programme (9 quarters)		Before programme	After programme	Difference
Results-based financing	1.33	1 694 470	6 991 502		0.26	0.59	0.33
Input-based financing	1.26	3 097 135	8 489 457		0.50	0.77	0.27
Control	1.40	2 924 591	8 062 629		0.42	0.64	0.22

US$: United States dollars.

Notes: Results-based financing facilities received increased funding tied to performance on pre-agreed indicators; input-based financing facilities received increased funding not tied to performance; control facilities were under the usual funding system without additional financing in Zambia. There were 175, 173 and 175 primary health-care facilities in the results-based financing, input-based financing and control groups, respectively. Costs of consumables (e.g. drugs and supplies) were obtained from a data set compiled by Medical Stores Limited, the national pharmaceutical distribution hub, from January 2011 through June 2014.

**Table 2:** Incremental costs of consumables under three methods of health-care financing before and after implementation of the results-based financing programme in Zambia, April 2012 to June 2014

Group comparisons	Population in millions	Difference in differences, US$	
		Per quarter per capita	Per capita over 9 quarters
Results-based financing versus control	1.33	+0.11	+0.97
Results-based financing versus input-based financing	1.26	+0.06	+0.57
Input-based financing versus control	1.40	+0.04	+0.40

US$: United States dollars.

Notes: Results-based financing facilities received increased funding tied to performance on pre-agreed indicators; input-based financing facilities received increased funding not tied to performance; control facilities were under the usual funding system without additional financing in Zambia.

**Fig. 4:**
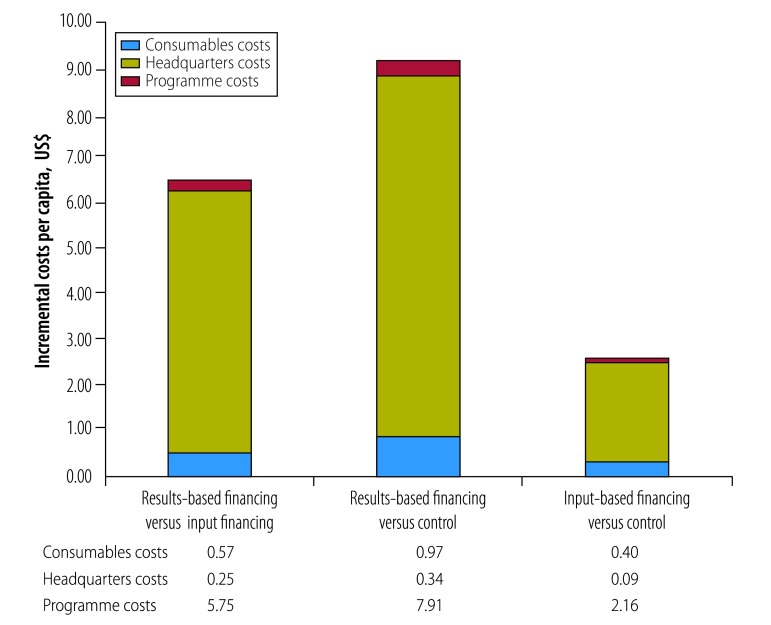
Incremental costs per capita of three methods of health-care financing in Zambia, April 2012 to June 2014

Fig. 4 summarizes the overall incremental costs per capita during the programme implementation period. Costs in the results-based financing group were US$ 6.56 and US$ 9.21 per capita more than in the input-based financing and the control groups, respectively. In input-based financing group, costs were US$ 2.66 per capita more than in the control group.

US$: United States dollars.

Notes: Results-based financing facilities received increased funding tied to performance on pre-agreed indicators; input-based financing facilities received increased funding not tied to performance; control facilities were under the usual funding system without additional financing in Zambia. Costs of consumables (e.g. drugs and supplies) were obtained from a data set compiled by Medical Stores Limited, the national pharmaceutical distribution hub, from January 2011 through June 2014.

Table 3 shows coverage of the key maternal health services and quality of care of services at baseline and endline from the household or health-facility surveys.^16^ A statistically significant difference was found in both *Haemophilus influenza* type B vaccination and use of injectable contraceptives, where the results-based financing group had higher use than the input-based financing group. The uptake of injectable contraceptives, for example, increased from 6.5% to 34.0% and from 9.9% to 15.6% in the results-based financing and input-based financing groups, respectively. Compared with the control group, the provision of services increased in the results-based financing group by 12.8% for institutional deliveries, 8.2% for postnatal care, 19.5% for injectable contraceptives and from 3.0% to 20.4% for vaccinations. The input-based financing group had significantly greater improvements in institutional deliveries and postnatal care versus the control group.

**Table 3:** Coverage and quality of key maternal and child health services in districts participating in the cluster randomized trial on results-based financing, Zambia, April 2012 to June 2014

Service	Before programme				After programme				Difference in differences^a^		
	Results-based financing group	Input-based financing group	Control group		Results-based financing group	Input-based financing group	Control group		Results-based financing versus input-based financing	Results-based financing versus control	Input-based financing versus control
**Coverage of key maternal and child services, %**											
Institutional deliveries	67.7	56.1	70.5		82.2	75.7	72.1		-4.9	+12.8***	+17.5***
Antenatal care	96.8	95.7	95.9		98.8	99.3	99.3		-1.5	-1.5	0.0
Postnatal care	69.2	55.7	75.8		83.6	73.3	81.9		-5.1	+8.2**	+13.2***
BCG vaccine	95.6	97.8	97.6		100.0	99.5	95.7		+3.1	+7.0*	+2.8
Diphtheria, pertussis and tetanus vaccine	97.1	95.2	95.8		98.6	97.5	91.9		-1.0	+6.1*	+5.6
*Haemophilus influenza* type B vaccination	82.5	88.3	81.8		97.9	88.7	78.1		+18.6***	+20.4***	+0.3
Intermittent preventive treatment	92.0	92.4	95.1		98.0	96.1	98.1		+2.3	+3.0**	+0.7
Injectable contraceptives^b^	6.5	9.9	7.7		34.0	15.6	15.7		+21.8**	+19.5**	-2.3
**Quality index^c^ of key maternal and child services, %**											
Institutional deliveries	65.5	66.8	67.0		73.5	74.1	71.9		+0.7	+3.1	+2.4
Antenatal care	66.9	69.1	68.6		75.0	77.2	73.8		+0.0	+2.9	+2.8
Postnatal care	66.7	68.4	68.3		74.1	76.6	73.4		-0.8	+2.3	+3.0
Vaccination	78.7	80.7	81.7		81.2	80.0	80.4		+3.2	+3.8	+0.6
Injectable contraceptives	77.7	78.6	80.6		81.6	77.6	74.8		+4.9	+9.7	+4.8

BCG: Bacillus Calmette–Guérin.

* *P* < 0.10; **P < 0.05; ***P < 0.01.

^a^ The impact was estimated with linear probability models with difference-in-difference specification, including controls for district stratification. Errors are clustered at the community level.

^b^ Data were from the health-facility survey.

^c^ Quality index was constructed at the group level, through averages of key quality dimensions across-facilities, and thus no statistical test was conducted.

Notes: Results-based financing facilities received increased funding tied to performance on pre-agreed indicators; input-based financing facilities received increased funding not tied to performance; control facilities were under the usual funding system without additional financing in Zambia.

Quality-of-care indices in the results-based financing group increased more than in the input-based financing group for institutional deliveries (0.7%), vaccinations (3.2%) and injectable contraceptives (4.9%; Table 3). Compared with the control group, the results-based financing group had greater improvements in quality of care: by 3.1% for institutional deliveries, 2.9% for antenatal care, 2.3% for postnatal care, 3.8% for vaccinations and 9.7% for injectable contraceptives. The input-based financing group also had improved quality of care in comparison with the control group.

Among the total 1.33 million population, 11 more lives were saved among pregnant women and 214 more lives among children younger than 5 years in the results-based financing group compared with the input-based financing group over the programme implementation period (without including quality of care in the analysis; Table 4). In the results-based financing group, 22 lives were saved among pregnant women and 497 lives among children younger than 5 years compared with the control group. After adjustment for improved quality of care, the estimated number of lives saved in the results-based financing group doubled. Specifically, 279 more lives were saved among mothers and children (lower–upper bounds: 214–324) in the results-based financing group versus the input-based financing group, and 641 more lives were saved (lower–upper bounds: 580–700) versus the control group.

**Table 4:** Lives saved among mothers and children in districts participating in the cluster randomized trial on results-based financing, Zambia, April 2012 to June 2014

Population	No. of deaths estimated by LiST						No. of lives saved, quality unadjusted				No. of lives saved, quality adjusted		
	Results-based financing group	Input-based financing group	Control group	Input-based financing, quality adjusted	Control, quality adjusted		Results-based financing versus input-based financing	Results-based financing versus control	Input-based financing versus control		Results-based financing versus input-based financing	Results-based financing versus control	Input-based financing versus control
**Children < 5 years**													
Year 2013	4478	4537	4636	4553	4673		59	158	99		75	195	120
Year 2014	4334	4489	4673	4524	4752		155	339	184		190	418	228
Subtotal	8812	9026	9309	9077	9425		214	497	283		265	613	348
**Maternal deaths**													
Year 2013	141	145	149	146	151		4	8	4		5	10	5
Year 2014	133	140	147	142	151		7	14	7		9	18	9
Subtotal	274	285	296	288	302		11	22	11		14	28	14
**Total lives saved (point estimate)**	NA	NA	NA	NA	NA		225	519	294		279	641	362
Lower bound	NA	NA	NA	NA	NA		167	461	226		214	580	293
Upper bound	NA	NA	NA	NA	NA		267	576	356		324	700	430

LiST: Lives Saved Tool; NA: not applicable.

Notes: Results-based financing facilities received increased funding tied to performance on pre-agreed indicators; input-based financing facilities received increased funding not tied to performance; control facilities were under the usual funding system without additional financing in Zambia. The quality-adjusted results considered the impact of the programme on quality of care. All the calculations in this table used the standard baseline population of 1.33 million and baseline coverage of health services of the quality-adjusted coverage in the results-based financing group.

When converting health benefits to QALYs gained, 5325 QALYs were saved (lower– upper bounds: 3948–6317) in the results-based financing group versus the input-based financing group, and 12 291 QALYs (lower–upper bounds: 10 905–13 639) versus the control group, when quality was not adjusted (Table 5). These were equivalent to gaining 0.0041 QALYs and 0.01 QALYs per capita. The number of QALYs gained was doubled when the improvement of quality of care was considered.

**Table 5:** Quality-adjusted life years among mothers and children in the results-based financing programme in Zambia, April 2012 to June 2014

Population	QALYs gained, mid-point (lower-upper bounds)					
	Results-based financing versus input-based financing		Results-based financing versus control		Input-based financing versus control	
	Quality-unadjusted	Quality-adjusted	Quality-unadjusted	Quality-adjusted	Quality-unadjusted	Quality-adjusted
Children < 5 years	5 088 (3 733–6 015)	6 300 (4 826–7 323)	11 816 (10 480–13 100)	14 574 (13 195–15 953)	6728 (5 171–8 131)	8 274 (6 704–9 843)
Pregnant women	237 (216–302)	302 (237–345)	475 (425–539)	604 (539–626)	237 (176–302)	302 (237–345)
All	5 325 (3 948–6 317)	6 602 (5 064–7 688)	12 291 (10 905–13 639)	15 178 (13 734–16 579)	6 966 (5 347–8 433)	8 576 (6 942–10 188)

QALY: quality-adjusted life year.

Notes: Results-based financing facilities received increased funding tied to performance on pre-agreed indicators; input-based financing facilities received increased funding not tied to performance; control facilities were under the usual funding system without additional financing in Zambia. The estimated bounds assume independence of the eight maternal and child health indicators included in the analysis. The quality-adjusted results considered the impact of the programme on quality of care. All the calculations in this table used the standard baseline population of 1.33 million and baseline coverage of health services of the quality-adjusted coverage in the results-based financing group.

The incremental cost–effectiveness ratios of results-based financing were US$ 1642 and US$ 999 per QALY gained when compared with the input-based financing and control groups, respectively, without adjustment for quality of care (Table 6). These ratios fell to US$ 1324 and US$ 809 per QALY gained if quality of care was included. The incremental cost–effectiveness ratios for the input-based financing versus the control group were US$ 508 and US$ 413 per QALY gained, without and with quality adjustment, respectively.

**Table 6:** Incremental cost–effectiveness ratios of three methods of health-care financing in the results-based financing programme, Zambia, April 2012 to June 2014

Group comparisons	Incremental cost–effectiveness ratios, mid-point (lower-upper bounds)			
	Cost per life saved, quality unadjusted, US$	Cost per QALY gained, quality unadjusted, US$	Cost per life saved, quality adjusted, US$	Cost per QALY gained, quality adjusted, US$
Results-based financing versus input-based financing	38 857 (32 744–52 351)	1 642 (1 384–2 214)	31 336 (26 983–40 853)	1 324 (1 141–1 727)
Results-based financing versus control	23 666 (21 324–26 643)	999 (900–1 126)	19 161 (17 546–21 177)	809 (741–895)
Input-based financing versus control	12 040 (9 943–15 663)	508 (419–662)	9 999 (8 232–12 081)	413 (348–510)

QALY: quality-adjusted life year; US$: United States dollars.

Notes: Results-based financing facilities received increased funding tied to performance on pre-agreed indicators; input-based financing facilities received increased funding not tied to performance; control facilities were under the usual funding system without additional financing in Zambia. The quality-adjusted results considered the impact of the programme on quality of care.

## Discussion

This study was an attempt to estimate the value of results-based financing on both costs and effectiveness of maternal and child care. The results showed that the results-based financing programme in Zambia, in comparison with the control group, is a cost–effective approach to improving maternal and child health. The mid-point incremental cost–effectiveness ratios were US$ 999 and US$ 809 per QALY gained, without and with quality adjustment, respectively. Both values were less than 1.5 times the GDP per capita in 2013 in Zambia.^29^ In comparison with input-based financing, there were greater health benefits in results-based financing districts, but with higher costs. However, the results-based financing programme remained cost–effective using the same threshold, and the mid-point incremental cost–effectiveness ratio was US$ 1324 per QALY gained.

This study is consistent with many studies showing a favourable impact of results-based financing in improving the uptake of maternal and child health services.^8^^,^^10^ We found that the major increases in quantities of health services were institutional deliveries, postnatal care visits, *Haemophilus influenza* type B vaccination and family planning using injectable contraceptives. The potential improved motivation among staff, greater flexibility in managing financial resources and strengthened supervision through results-based financing may contribute to such improvements. Concerns have been raised whether results-based financing neglects non-incentivized services.^11^ The chance of such an effect in our study seems slim given that the incentivized indicators spanned the majority of services provided in a health facility. In Haiti, a study showed no negative effect of results-based financing on non-incentivized services.^11^

Compared with the control districts, facilities in results-based financing districts showed substantial improvements in using injectable contraceptives for family planning. The improvement of injectable contraceptives is an important factor in determining the cost–effectiveness ratio. The results-based financing programme spent about 29% of incentive payments on family planning. Investment in family planning is regarded as one of most cost–effective approaches to reducing both maternal and child mortality rates,^19^^,^^30^^,^^31^ not only avoiding unintended pregnancies, but also reducing health risks for both pregnant women and infants themselves.

Few studies have examined increased financing alone against the usual financing method without additional funding. Compared with control, the input-based financing method was cost–effective, with a lower incremental cost–effectiveness ratio than results-based financing. In health facilities where resources are constrained, this suggests that simply providing more financial support would improve maternal and child health substantially. However, input-based financing also contained a directive that these funds be spent on maternal and child health services. This signalling may have played an important role in coordinating efforts around the additional resources for maternal and child health at the district and facility level. As none of the input-based financing resources were allowed to be spent as staff bonuses, the amount of financing available for facility investments was similar to that with results-based financing after subtracting staff bonuses (47% of results-based financing transfers to facilities were allocated to staff bonuses).

We incorporated quality of care in the results-based financing cost–effectiveness modelling. Given that quality of care is one of the major components of the programme and a component of the payment formula to health facilities, an analytical model not incorporating quality of care would underestimate the cost–effectiveness of results-based financing. Studies show that improving quality of care is important for achieving better health outcomes and could greatly reduce mortality rates.^32^^–^^34^

Services with high baseline coverage, such as antenatal care and vaccination, showed the least improvement under results-based and input-based financing. Results-based financing continued incentivizing those services. As a means to enhance the cost–effectiveness of the programme, payments to services with high coverage may need to be reduced. Reducing excessive verification costs could be another approach to improve the efficiency of the programme. In Zimbabwe, for better efficiency, a risk-based verification approach was implemented.^35^ Cost–effectiveness is not the only criteria for decision-making. In scaling up the results-based financing programme, other aspects need to be considered, such as affordability, spillover effects, sustainability, equity and political factors.^27^

This study has several limitations. First, the focus of the study was financial costs instead of economic costs. Economic costs in the results-based financing districts may be slightly higher than those in other districts, as the staff members tend to be more satisfied with their jobs and work additional time. Second, converting the coverage of health services to health benefits relied on both the international literature about the effectiveness of the interventions and the overall national statistics in Zambia. These sources do not necessarily represent the parameters in the results-based financing intervention areas in this study. Third, the CIs of incremental cost–effectiveness ratios assumed that the indicators analysed are statistically independent; as costs and services are often positively correlated, these CIs should be regarded as the outer limits of the true intervals. Fourth, payments to input-based financing facilities deviated from the original plan. Only 56% of the results-based financing was transferred to input-based financing districts, adding complexity to the comparisons. Fifth, the cost estimates may have included certain cost categories such as introductory training that are generally present in pilot programmes, but not mature programmes. This element would overstate the costs of results-based financing against the alternatives. Sixth, the baseline for some indicators is not well-balanced. The control districts had a relatively high coverage of some maternal and child health services when compared with input-based financing. Lastly, the sample population for the household survey was from households with recent births, and some measures, such as family planning, were estimated from health-facility data to mitigate the potential bias.

This study provides empirical evidence on cost–effectiveness of results-based financing using a rigorous randomized trial. Both quantity and quality improvements contributed to the cost–effectiveness of incentivized financing. The analysis compared results-based financing with an input-based financing method, demonstrating the pure impact of incentives alongside associated activities while controlling for total financial flows. Additionally, this study presented an approach to model the impact of quality improvement on health outcomes through a Delphi panel process.
